# Supplementary material for: Informed and Empowered: A Pre–Post Evaluation of a Whiteboard Video for Sexual Health Education in Female Adolescents and Young Adults with Cancer
Source: Curr Oncol. 2025 Dec 1;32(12):681. doi: 10.3390/curroncol32120681 (PMC12731592; doi:10.3390/curroncol32120681)
Supplement: Supplementary file 1 [file curroncol-32-00681-s001.zip › curroncol-3929412-supplementary.pdf]

Data Supplement S1. Mapping of Video Educational Content to Evidence Sources

Theme	Summary of Core Video Message	Representative Evidence Sources
**1. Safety and Myths About Intimacy During Cancer Treatment**	The video clarifies that sexual activity is generally safe during cancer treatment but may need to be modified or temporarily avoided when infection or bleeding risk is high. It reinforces that cancer itself is not contagious and cannot be transmitted through intimacy.	Cancer Care Ontario. *Intimacy and Sex: For people with cancer and their partners.* 2020.<br>ASCO Clinical Practice Guideline: *Interventions to Address Sexual Problems in People With Cancer.* J Clin Oncol 2018.<br>American Cancer Society. *Is Cancer Contagious?* 2021.<br>National Cancer Institute. *Common Cancer Myths and Misconceptions.* 2021.<br>BC Cancer. *Sexual Health.* 2021.
**2. Physical Effects of Cancer Treatment on Sexual Health**	The video explains that chemotherapy, radiation, and hormonal therapy may induce premature menopause, vaginal dryness, pain, and reduced sexual desire. It normalizes these physical effects and encourages medical discussion.	North American Menopause Society (NAMS). *2020 Position Statement on Genitourinary Syndrome of Menopause.*<br>NCCN Clinical Practice Guidelines in Oncology: *Survivorship*.<br>Sears CS et al. *A Comprehensive Review of Sexual Health Concerns After Cancer Treatment.* Eur J Cancer Care 2018.<br>American Cancer Society. *Women: Managing Cancer-Related Sexual Problems.* 2021.<br>BC Cancer. *Sexual Health.* 2021.
**3. Management of Vaginal Dryness and Pain**	The video describes strategies for vaginal dryness and discomfort, including use of non-hormonal moisturizers and lubricants. It explains differences among water-, silicone-, and oil-based products with respect to condom and sex-toy compatibility, and notes that local estrogen therapy may be considered in consultation with oncology.	North American Menopause Society (NAMS). *2020 GSM Position Statement.*<br>ACOG Committee Opinion No. 659 (2016): *Use of Vaginal Lubricants and Moisturizers in Women With Dyspareunia.*<br>Kingsberg SA et al. *Vaginal Moisturizers and Lubricants: A Review of Products, Composition, and Efficacy. Climacteric* 2019.<br>Cochrane Database Syst Rev 2014: *Vaginal Dilator Therapy for*

		Women Receiving Pelvic Radiotherapy.<br>American Cancer Society. *Women: Managing Cancer-Related Sexual Problems.* 2021.<br>BC Cancer. *Sexual Health.* 2021.
**4. Rehabilitation and Pelvic Floor Therapy**	The video introduces pelvic-floor physiotherapy as a helpful strategy for dyspareunia and pelvic discomfort following treatment, emphasizing that physical rehabilitation can complement medical and psychological support.	Ghaderi F et al. *Pelvic Floor Rehabilitation in the Treatment of Women with Dyspareunia: A Randomized Controlled Clinical Trial. Int Urogynecol J* 2019.<br>ASCO Guideline: *Interventions to Address Sexual Problems in People With Cancer. J Clin Oncol* 2018.<br>NCCN *Survivorship* Guidelines v2.2021.
**5. Psychosocial and Communication Supports**	The video acknowledges that sexual health is influenced by emotional well-being, relationships, and communication. It encourages patients to seek counseling or therapy, talk openly with partners and providers, and view sexual recovery as part of holistic survivorship.	ASCO Guideline: *Interventions to Address Sexual Problems in People With Cancer. J Clin Oncol* 2018.<br>NCCN *Survivorship* Guidelines.<br>Bober SL, Kingsberg SA, Faubion SS. *Sexual Function After Cancer: Paying the Price of Survivorship. J Clin Oncol* 2020.<br>American Cancer Society. *Women: Managing Cancer-Related Sexual Problems.* 2021.<br>BC Cancer. *Sexual Health.* 2021.
**6. Contraception and STI Protection During Treatment**	The video advises the use of contraception and barrier protection (e.g., condoms, dental dams) during and after treatment to prevent pregnancy and sexually transmitted infections, especially when immune function may be compromised.	Cancer Care Ontario. *Intimacy and Sex: For people with cancer and their partners.* 2020.<br>NCCN *Survivorship* Guidelines.<br>American Cancer Society. *Women: Managing Cancer-Related Sexual Problems.* 2021.<br>BC Cancer. *Sexual Health.* 2021.

Data Supplement S2. Patient Education Materials Assessment Tool for Audiovisual Materials Scoring

	Reviewer					
Item	SC	CV	AR	AB	CV	JF
**Content**	1	1	1	1	1	1
The material makes its purpose completely evident	1	1	1	1	1	1
The material does not include information or content that distracts from its purpose	1	1	1	1	1	1
**Words Choice & Style**						
The material uses common, everyday language	1	1	1	1	1	1
Medical terms are used only to familiarize audience with the terms; When used, medical terms are defined	1	1	1	1	1	1
The material uses the active voice	1	1	1	1	1	1
**Numbers**						
Numbers appearing in the material are clear and easy to understand	1	1	N/A	N/A	N/A	N/A
The material does not expect the user to perform calculations	1	1	1	1	1	1
**Organization**	1	1	1	1	1	1
The material breaks or “chunks” information into short sections	1	1	1	1	1	1
The material’s sections have informative headers	0	1	1	1	1	1
The material presents information in a logical sequence	1	1	1	1	1	1
The material provides a summary	1	1	1	0	0	1
**Layout and design**						
The material uses visual cues to draw attention to key points	1	1	1	1	1	1
Text on the screen is easy to read	1	1	1	1	1	1
The material allows the user to hear the words clearly	1	1	1	1	1	1
**Use of visual aids**						
The material uses visual aids whenever they could make it easier to act on the instructions	1	1	1	1	1	1

The material's visual aids reinforce rather than distract from content	1	1	1	1	1	1
The material's visual aids have clear titles or captions	1	1	0	0	1	1
The material uses illustrations and photographs that are clear and uncluttered	1	1	1	1	1	1
The material uses simple tables with short and clear row and column headings	N/A	N/A	N/A	N/A	N/A	N/A
**Actionability**						
The material clearly identifies at least one action the user can take	1	1	1	1	1	1
The material addresses the user directly when describing actions	1	1	1	1	1	1
The material breaks down any action into manageable, explicit steps	1	1	1	1	1	1
The material provides a tangible tool whenever it could help the user take action	1	0	1	0	1	0
The material provides simple instructions or examples of how to perform calculations	N/A	N/A	N/A	N/A	N/A	N/A
The material explains how to see the charts, graphs, tables, or diagrams to take actions	N/A	N/A	N/A	N/A	N/A	N/A
The material uses visual aids whenever they could make it easier to act on the instructions	1	1	1	1	1	1
Understandability, %	100	94	94	94	94	100
Actionability, %	80	80	100	80	100	80

Data Supplement S3. Examples of Participant Quotations by Theme

Theme	Relevant quotations
Clarity & Accessibility<br>• Non-judgmental language<br>• Visuals reinforced learning	"Talking through the language of sexual health can be helpful and equip patients with enough information to be able to do their own research or engage with their healthcare team. it was very neutral and non-judgemental, and I think importantly emphasizes the help you get from self-exploration during this time."<br><br>"How clearly it explained sexual side effects and ways to manage them"<br><br>"I like all the visuals in the videos especially of the vagina and the captions"<br><br>"The visuals and breakdown of what is happening. I didn't get this info while in treatment."<br><br>"Knowing that being sexually active is allowed during chemotherapy, the different types of lubricants that can be used and the diagrams of body parts."
Normalization<br>• Reduced stigma<br>• Encouraged provider conversations	"The fact that it normalized talking to health care professionals about sexual health"<br><br>"I appreciated the destigmatisation of discussing sexual health with a medical team, it's easy to feel awkward about it but the video explained that there is no shame in it."<br><br>"Normalized sexual health issues and made me feel like there were lots of options"<br><br>"The fact that it normalized talking to health care professionals about sexual health"<br><br>"Validation that cancer treatment can cause a variety of things that affect your sexual health"<br><br>"Feeling seen and having a better understanding of my body"
Content Gaps	"How to talk to your partner"

• Partner communication<br>• Psychosocial impact<br>• Cancer-specific information	"Tips of communication with partner and/or healthcare team about sexual health"<br><br>"I think a big part of sex post diagnosis that should be discussed is consent, and how that relates to a decreased sex drive. I think there is an immense importance on stressing that you do not owe anyone sex ever, but especially if your sex drive has gone down due to illness. There is very little media that focuses on how to discuss the changes in sexual interest with your partner, and I think that those discussions need to be had. A conversation on consent, how to balance caregiving and sexual desire, and how to discuss sexual wellness post treatment with a partner, long or short term."<br><br>"Adding a section on how to discuss sexual wellness post diagnosis with a partner, and using less distracting background music."<br><br>"Tips on how to initiate a discussion with your partner around changes in sexual arousal/desire"<br><br>"Maybe dealing with others expectations around sex. Like setting boundaries"<br><br>"Talk about other topics such as hair loss and mental health during chemo"<br><br>"I don't think anything is missing other than more emphasis on psychosocial aspects during treatment and as a survivor."<br><br>"It might be useful to acknowledge more of the psychosocial effects (fear, shame, confusion, frustration)."

	"Include symptoms such as hot flashes and changes in hormone levels, which can sometimes lead to weight gain or acne."<br><br>"I think discussing more specific impacts of specific treatments at least briefly could be useful. although speaking to your medical team is best to find out about what's best for your specific scenario, just a quick rundown of how different treatments have different impacts could be helpful"<br><br>"A little more in depth on side effects of treatments"<br><br>"Be mindful of patients with CNS cancers (brain/spine) as these can have whole other effects on sexual health and are often not discussed!"
Suggested Improvements<br>• Shorter length<br>• Better visuals<br>• Different narrator	"The video felt a bit too long"<br><br>"Could be shorter clips instead"<br><br>"Slightly shorter video would be nice"<br><br>"The video could have been shorter, the same music in the background for 13 minutes is not ideal but consistent with other educational video"<br><br>"I personally prefer videos that are shorter / more concise - under 10 mins?"<br><br>"Video is quite long, potential to break it up into chapters/inbed subsections in the youtube code for easier navigation. Could also potentially break it up into a series of shorter videos as information was tough to retain with brain fog/shorter attention span"

	"More images."<br><br>"More visually appealing, shorter"<br><br>"Making the visuals more engaging, some were not very relevant"<br><br>"Shorten length time, if video is geared towards teens and young adults the voice over should try and represent that age group."<br><br>"The tone of the narrators voice felt very much like an infomercial or advertisement- I kept expecting her to try to sell me something. The music was also very loud and distracting and took away from the overall educational content. I ended up having to turn on subtitles and turn down the volume to watch."<br><br>"Could it be narrated by physician who specializes in this topic? Or maybe an AYA patient or peer support person?"<br><br>"It could be narrated and introduced by a real doctor"

Data Supplement S4. Item-level pre- and post-video knowledge accuracy (N = 90)

No.	Knowledge Item (Topic)	Pre N (%)	Post N (%)	Δ (pp)
1	After receiving chemotherapy, how long should you wait until it is safe to kiss your partner?	46 (51%)	62 (69%)	+18
2	I can pass on my cancer to my partner by having sex with them.	85 (94%)	86 (96%)	+2
3	Cancer treatment can sometimes cause decrease in estrogen levels. Which of the following may happen with decreased estrogen levels?	78 (87%)	85 (94%)	+7
4	It is always safe to have sex during chemotherapy?	62 (69%)	71 (79%)	+10
5	You should wait for your doctor to bring up the topic of sexual health.	76 (84%)	85 (94%)	+10
6	Cancer treatment might make	75 (83%)	86 (96%)	+13

	you lose your interest in having sex.			
7	Vaginal moisturizers can be used to manage vaginal dryness.	62 (69%)	87 (97%)	+28
8	Which of the following types of lubricants are not safe to use with condoms?	24 (27%)	66 (73%)	+46
9	Which of the following can help manage vaginal dryness?	40 (44%)	78 (87%)	+43

Note. Δ (pp) indicates the absolute change in percentage points from pre- to post-video.
